# An Asian multi-national multi-institutional retrospective study comparing intracavitary versus the hybrid of intracavitary and interstitial brachytherapy for locally advanced uterine cervical carcinoma

**DOI:** 10.1093/jrr/rrac014

**Published:** 2022-04-22

**Authors:** Naoya Murakami, Ken Ando, Masumi Murata, Kazutoshi Murata, Tatsuya Ohno, Tomomi Aoshika, Shingo Kato, Noriyuki Okonogi, Anneyuko I Saito, Joo-Young Kim, Yasuo Yoshioka, Shuhei Sekii, Kayoko Tsujino, Chairat Lowanichkiattikul, Poompis Pattaranutaporn, Yuko Kaneyasu, Tomio Nakagawa, Miho Watanabe, Takashi Uno, Rei Umezawa, Keiichi Jingu, Ayae Kanemoto, Masaru Wakatsuki, Katsuyuki Shirai, Hiroshi Igaki, Jun Itami

**Affiliations:** Department of Radiation Oncology, National Cancer Center Hospital, Tokyo 104-0045, Japan; Department of Radiation Oncology, Gunma Prefectural Cancer Center, Gunma 373-8550, Japan; Department of Radiation Oncology, Gunma University Graduate School of Medicine, Gunma 371-8511, Japan; Department of Radiation Oncology, Gunma Prefectural Cancer Center, Gunma 373-8550, Japan; Department of Radiation Oncology, Gunma University Graduate School of Medicine, Gunma 371-8511, Japan; QST Hospital, National Institutes for Quantum Science and Technology, Chiba 263-8555, Japan; Department of Radiation Oncology, Gunma University Graduate School of Medicine, Gunma 371-8511, Japan; Department of Radiation Oncology, Saitama Medical University International Medical Center, Saitama 350-1298, Japan; Department of Radiation Oncology, Saitama Medical University International Medical Center, Saitama 350-1298, Japan; QST Hospital, National Institutes for Quantum Science and Technology, Chiba 263-8555, Japan; Department of Radiation Oncology, Juntendo University Faculty of Medicine, Tokyo 113-8431, Japan; Department of Radiation Oncology, National Cancer Center, Goyang 410-769, Korea; Radiation Oncology Department, Cancer Institute Hospital of Japanese Foundation for Cancer Research, Tokyo 135-8550, Japan; Department of Radiation Oncology, Hyogo Cancer Center, Hyogo 673-8558, Japan; Department of Radiation Therapy, Kita-Harima Medical Center, Hyogo 675-1392, Japan; Department of Radiation Oncology, Hyogo Cancer Center, Hyogo 673-8558, Japan; Division of Radiation Oncology, Department of Radiology, Faculty of Medicine Ramathibodi Hospital, Mahidol University, Bangkok 73170, Thailand; Division of Radiation Oncology, Department of Radiology, Faculty of Medicine Ramathibodi Hospital, Mahidol University, Bangkok 73170, Thailand; Department of Radiation Oncology, National Hospital Organization Fukuyama Medical Center, Hiroshima, Japan; Department of Radiation Oncology, National Hospital Organization Fukuyama Medical Center, Hiroshima, Japan; Department of Radiology, Chiba University Hospital, Chiba 260-8677, Japan; Department of Radiology, Chiba University Hospital, Chiba 260-8677, Japan; Department of Radiation Oncology, Tohoku University Graduate School of Medicine, Miyagi 980-8574, Japan; Department of Radiation Oncology, Tohoku University Graduate School of Medicine, Miyagi 980-8574, Japan; Department of Radiation Oncology, Niigata Cancer Center Hospital, Niigata 951-8566, Japan; QST Hospital, National Institutes for Quantum Science and Technology, Chiba 263-8555, Japan; Department of Radiology, Jichi Medical University Hospital, Tochigi 329-0498, Japan; Department of Radiology, Jichi Medical University Hospital, Tochigi 329-0498, Japan; Department of Radiation Oncology, National Cancer Center Hospital, Tokyo 104-0045, Japan; Department of Radiation Oncology, National Cancer Center Hospital, Tokyo 104-0045, Japan

**Keywords:** uterine cervical cancer, hybrid of intracavitary and interstitial brachytherapy (HBT), combined intracavitary/interstitial brachytherapy, image-guided adaptive brachytherapy

## Abstract

This study is an international multi-institutional retrospective study comparing the clinical outcomes between intracavitary brachytherapy (ICBT) and the hybrid of intracavitary and interstitial brachytherapy (HBT) for locally advanced cervical cancer patients treated with definitive radiation therapy. Locally advanced cervical cancer, the initial size of which is larger than 4 cm and treated by concurrent chemoradiotherapy and image-guided adaptive brachytherapy, were eligible for this retrospective study. Patients who received HBT at least once were included in the HBT group, and patients who received only ICBT were included in the ICBT group. Anonymized data from 469 patients from 13 institutions in Japan, one from Korea and one from Thailand, were analyzed. Two hundred eighty and 189 patients were included in the ICBT group and the HBT group, respectively. Patients in the HBT group had more advanced stage, non-Scc histopathology, a higher rate of uterine body involvement, larger tumor at diagnosis, larger tumor before brachytherapy and a lower tumor reduction ratio. With a median follow-up of 51.3 months (2.1–139.9 months), 4-y local control (LC), progression-free survival (PFS) and overall survival (OS) for the entire patient population were 88.2%, 64.2% and 83%, respectively. The HBT group received a higher HR-CTV D_90_ than that of the ICBT group (68.8 Gy vs 65.6 Gy, *P* = 0.001). In multivariate analysis, the non-Scc histological subtype, HR-CTV D_95_ ≤ 60 Gy, reduction ratio ≤ 29% and total treatment time (TTT) ≥ 9 weeks were identified as the independent adverse prognostic factors for LC. Regarding LC, no difference was found between ICBT and HBT (4-y LC 89.3% vs 86.8%, *P* = 0.314). After adjustment for confounding factors by propensity score matching, no advantage of applying HBT was demonstrated regarding LC, PFS, or OS. Despite the fact that HBT patients had more adverse clinical factors than ICBT patients, HBT delivered a higher dose to HR-CTV and resulted in comparable LC.

## INTRODUCTION

The definitive radiotherapy for cervical cancer consists of pelvic external beam radiation therapy (EBRT) covering the primary tumor and regional drainage lymphatic area followed by intracavitary brachytherapy (ICBT) [1, 2]. ICBT involves inserting two ovoids or ring applicators into the vagina, and a tandem applicator is inserted into the uterine canal, and a high dose is delivered directly to the primary tumor located in the uterine cervix. After magnetic resonance imaging (MRI) or computed tomography (CT) images with the brachytherapy applicators in place became available for brachytherapy dose calculation, studies on the relationship between delivered dose and local control (LC) were published, and it was discovered that a favorable clinical outcome is expected when the tumor responds well to the preceding EBRT and the tumor is confined within the proximity of brachytherapy applicators. On the other hand, if a residual tumor still exists that is far from the brachytherapy applicators after EBRT, the possibility of developing local recurrences is high [3]. To address this issue, the concept of 3D image-guided adaptive brachytherapy (3D-IGABT) was advocated in Europe [1, 4] and the United States [5, 6], and favorable clinical outcomes were reported [3, 7, 8]. A French group conducted a non-randomized prospective clinical trial comparing 2D ICBT (2D-ICBT) and 3D-IGABT, demonstrating that 3D-IGABT was superior in terms of toxicity and efficacy [9]. As a result, 3D-IGABT is considered the standard brachytherapy technique in definitive radiation therapy for locally advanced cervical cancer.

It is widely accepted that multi-catheter interstitial brachytherapy (ISBT) can effectively treat large tumors [10, 11]. However, only a few institutions can perform ISBT because it requires expertise and human resources, patients must be in bed overnight with the needles in place for ISBT multiple sessions until all brachytherapy treatment is completed. Following that, the hybrid of intracavitary/interstitial brachytherapy (HBT) was introduced, with promising clinical results [12–14]. Along with intracavitary applicators, a few extra interstitial needles are inserted into the involved parametrium during HBT. Because this method is much easier to perform than multi-catheter ISBT, it is anticipated that many institutions will begin to perform HBT. Yoshida *et al.* [15] conducted a simulation analysis and found that tumors smaller than 4 × 3 × 3 cm can be treated with ICBT, while tumors larger than 5 × 4 × 4 cm can be treated by multi-catheter ISBT, and tumors in between can be treated by HBT. However, currently, there exist no guidelines indicating which tumor should be treated with which types of brachytherapy.

There has been no prospective clinical trial comparing ICBT and HBT to investigate the superiority of HBT over conventional ICBT since the concept of HBT in the field of gynecological brachytherapy was introduced in the field of gynecological brachytherapy. As a result, it is unclear whether a more invasive procedure such as HBT is truly beneficial for patients with locally advanced uterine cervical cancer when compared to conventional ICBT.

Our study group originally intended to conduct a randomized clinical trial that directly compared clinical outcomes between ICBT and HBT. However, because the superiority of HBT over ICBT is evident due to the dose coverage superiority of HBT over ICBT, approximately 30% of participants thought it was unethical to perform such a clinical trial. As a result, a multi-institutional retrospective study comparing clinical outcomes of patients treated with ICBT and HBT was planned to determine whether or not there is a clinical improvement after the introduction of HBT. If clinical equipoise between ICBT and HBT is observed, it is ethically permissible to conduct a phase III clinical trial comparing clinical results of ICBT and HBT. If this is not the case, a validating single-arm clinical trial should be performed. The aim of this multi-institutional retrospective study is to compare the clinical outcomes of ICBT and HBT for locally advanced uterine cervical cancer patients treated with definitive chemoradiotherapy.

## MATERIALS AND METHODS

Inclusion criteria for this study were as follows: previously untreated uterine cervical cancer patients who were 20 years old or older and were treated with definitive chemoradiotherapy between 2000 and 2016 with a follow-up period longer than 2 years were included in this retrospective study — patients who died within 2 years after treatment were also included. Before treatment, the initial maximum tumor size should be greater than 4 cm as measured by MRI. MRI should also be used to determine tumor size prior to brachytherapy. These two MRIs taken prior to chemoradiotherapy and brachytherapy were used to calculate the tumor reduction ratio. Brachytherapy dose calculation should be based either on CT or MRI (3D-IGABT). The following were the study’s exclusion criteria: (i) patients who had induction chemotherapy, hysterectomy, or history of pelvic irradiation, (ii) patients who had treatment for invasive cancer other than uterine cervical cancer within the previous 5 years, (iii) histopathology other than squamous cell carcinoma (Scc), adenocarcinoma, or adenosquamous cell carcinoma, (iv) patients with extra-pelvic disease extension except for 1–3 para-aortic lymph node(s) (PALN) less than 2 cm in size; the patients with 4 PALNs and/or larger than 2 cm PALN, and (v) patients who received only palliative doses less than 40 Gy or brachytherapy less than twice.

**Fig. 1.**
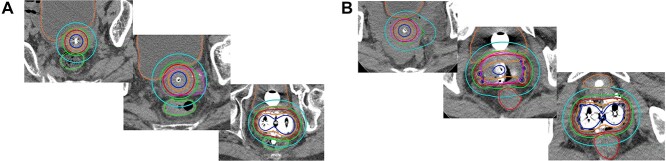
Shows an example of a comparison of dose distributions in T3b patients treated with ICBT (A) and HBT (B). The solid red line represents 100% isodose line (6 Gy), the dark blue line, 200% isodose line (12 Gy), the orange line, 150% (9 Gy), the green line, 80% (4.8 Gy), the sky blue line, 50% (3 Gy) and the pink line represents the HR-CTV, respectively. In Fig. 1A, even though the dwell time of the left ovoid was set longer than the dwell time of the right ovoid in order to cover as much of the left side parametrial extension as possible, the 100% isodose line could not adequately cover a large portion of the left parametrial extension. On the other hand, in Fig. 1B, since interstitial needles were inserted to cover bilateral parametrial extension, HR-CTV was well covered by the 100% isodose line.

The HBT group included patients who had received HBT at least once. For example, a patient who received HBT twice and experienced favorable tumor shrinkage and subsequently received ICBT twice was included in the HBT group. Figure 1 depicts an example of a comparison of dose distributions in T3b patients treated with ICBT and HBT.

The following clinical data were collected: patient’s age, the International Federation of Gynecology and Obstetrics (FIGO) stage (2008), histological subtypes, the presence of uterine body involvement, pyometra, parametrial invasion, or hydronephrosis, largest tumor size at diagnosis and before brachytherapy both assessed by MRI (cm), total treatment time (TTT) (week), dose of whole pelvis (WP) irradiation (Gy), dose of pelvic irradiation with a central shield (CS), dose of lymph node boost, systemic chemotherapy regimen, acute toxicities and late toxicities assessed by the Common Terminology Criteria for Adverse Events v4.0, and brachytherapy dosimetric parameters (high-risk clinical target volume [HR-CTV]; D_90_, D_95_, rectum D_2cc_ and bladder D_2cc_). While the majority of institutions participating in this study used 3D conformal radiation therapy with CS in the latter part of pelvic irradiation, some institutions use intensity modulated radiation therapy (IMRT). In IMRT, CTV included gross tumor volume, the uterine body, uterine cervix, parametrium and upper part of vagina. A planning target volume was created, adding adequate margins to CTV to compensate for organ motion, and no CS-like IMRT plan was applied. Therefore, the CTV dose was used as the central pelvic dose in this study. In this study, late adverse events of greater than grade 1 were counted as events. Because the dose contribution from CS to the primary site is difficult to assess [16], dose contribution only from WP was used in calculating the total dose of EBRT and brachytherapy and expressed in the form of the equivalent dose in 2 Gy fractions (EQD_2_) according to the linear-quadratic model [17].

The primary endpoint was the LC rate. The progression of the primary site was considered a local failure, and the LC rate was calculated from the start of the definitive radiation therapy to the date of local failure confirmation. Primary sites include the uterine cervix, uterine body, parametrium, or vagina in patients with initial vaginal invasion. Secondary endpoints included overall survival (OS), progression-free survival (PFS), and the late complication rate for the rectum, the bladder and the vagina. The Kaplan–Meier method was used to calculate survival curves. Univariate and multivariate analysis were used to investigate factors that influenced the recurrence or development of complications. Statistical significance was defined as a *P*-value of <0.05. Multivariate analysis using the Cox regression analysis was performed on factors with a *P*-value of <0.05. The hazard ratios were estimated using Cox proportional-hazards models. Baseline characteristics that were imbalanced between the two groups and had an influence on prognosis were used to perform 1:1 propensity score matching. Statistical analyses were conducted using IBM SPSS Statistics version 25.0 (SPSS, Inc., Chicago, IL) and EZR (XXX Medical Center, YYY University) [18], that is a freely available modified version of R (The R Foundation for Statistical Computing, Vienna, Austria) commander designed to add statistical functions frequently used in biostatistics.

## RESULTS

**Fig. 2.**
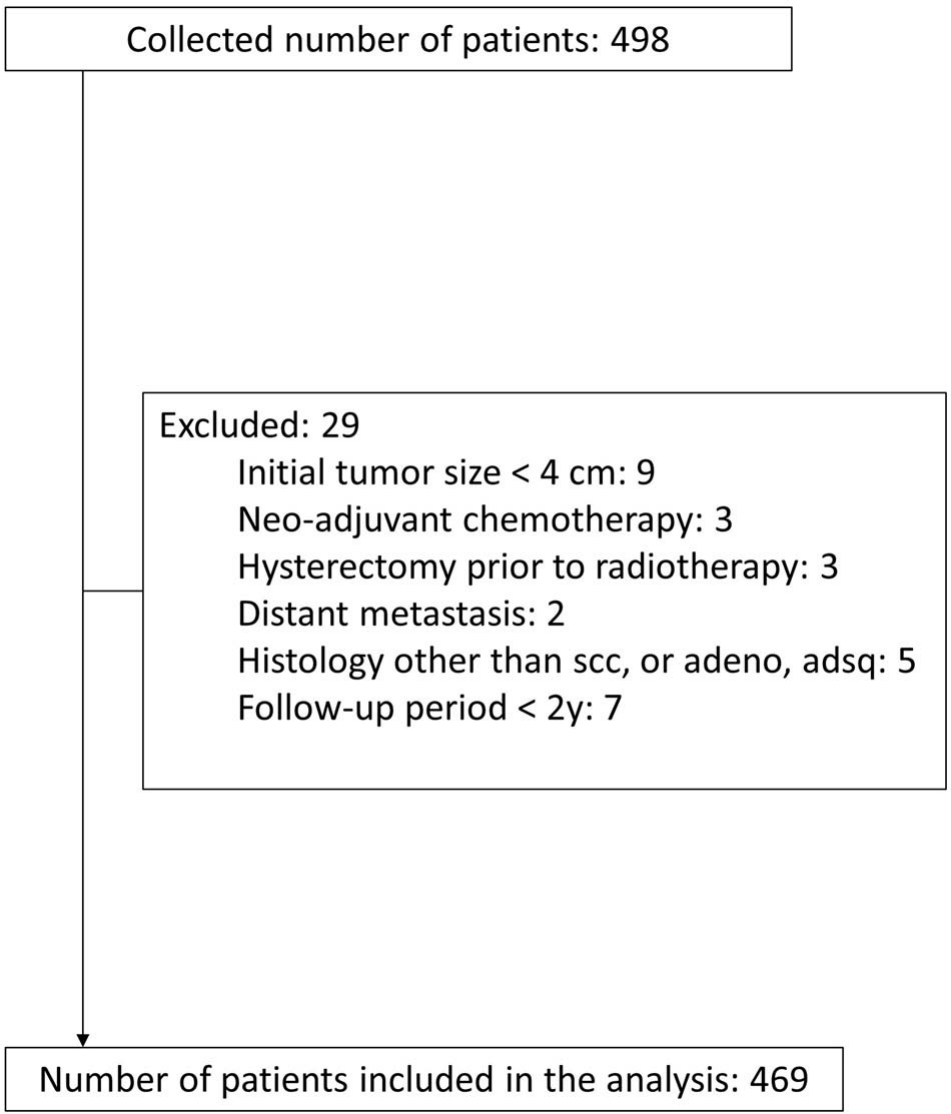
Shows the CONSORT flow diagram of the study.

Anonymized data sets from 498 patients were collected from 13 institutions in Japan, including those involved in the Working Group of the Gynecological Tumor Committee of the Japanese Radiation Oncology Study Group (JROSG), one from Korea and one from Thailand. Only one institution, which provided 10 patients’ data for this study, calculates brachytherapy doses based on MRI. The exclusion criteria resulted in the exclusion of 29 patients, leaving 469 patients in the analysis (Fig. 2). Table 1 shows patient characteristics. Two hundred eighty and 189 patients were included in the ICBT group and the HBT group, respectively. The ICBT group had a longer follow-up period than that of the HBT group, with statistical significance (60 months vs 45.5 months, *P* < 0.001). Patients in the HBT group had a more advanced stage, non-Scc histopathology, a higher rate of uterine body involvement, a larger tumor at diagnosis, a larger tumor before brachytherapy, and a lower tumor reduction ratio, implying that patients in the HBT group had poorer prognostic factors than patients in the ICBT group.

**Table 1:** Patient characteristics

		ICBT (*n* = 280)	HBT (*n* = 189)	*P-*value
Age (median), years		55 (26-86)	58 (26-81)	0.237
Follow-up period (median), months		55.3 (2.1-139.9)	44.6 (3.0-105.3)	<0.001^*^
FIGO stage (2008)				
	IB2-II	189 (67.5%)	75 (39.7%)	<0.001^*^
	III-IVA	91 (32.5%)	114 (60.3%)	
Histological subtypes				
	Squamous cell carcinoma	268 (95.7%)	170 (89.9%)	0.005^*^
	Adenocarcinoma	7 (2.5%)	17 (9%)	
	Adenosquamous carcinoma	5 (1.8%)	2 (1.1%)	
Uterine body invasion				
	Yes	90 (32.1%)	92 (48.7%)	<0.001^*^
	No	190 (67.9%)	96 (50.8%)	
	N/A	0 (0%)	1 (0.5%)	
Pyometra				0.140
	Yes	77 (27.5%)	148 (78.3%)	
	No	203 (72.5%)	41 (21.7%)	
Parametrium invasion				0.013^*^
	Yes	232 (82.9%)	171 (90.5%)	
	No	48 (17.1%)	18 (9.5%)	
Hydronephrosis				<0.001^*^
	Yes	38 (13.6%)	58 (30.7%)	
	No	242 (86.4%)	131 (69.3%)	
Pelvic LN metastasis				
	Yes	147 (52.5%)	106 (56.1%)	0.445
	No	133 (47.5%)	83 (43.9%)	
Tumor size at diagnosis (median, cm)		5.4 (4.0-12.0)	5.7 (4.1-14.5)	0.001^*^
Tumor size before brachytherapy (median, cm)		3.8 (0.0-6.6)	4.3 (2.0-10.3)	<0.001^*^
Reduction ratio (%)		31 (0-100)	25 (0-71)	<0.001^*^
Total treatment time (median, weeks)		7 (5-11)	7 (5-14)	0.600

FIGO: the International Federation of Gynecology and Obstetrics

LN: lymph node

ICBT: intracavitary brachytherapy

HBT: hybrid brachytherapy

^*^Statistical significance was defined as a *P*-value of <0.05.

Table 2 shows the treatment details. When compared to the HBT group, the patients in the ICBT group used more often WP alone as EBRT and fewer brachytherapy fractions compared to the HBT group, suggesting that large tumors in the ICBT group were treated primarily with EBRT and less brachytherapy was used, presumably because ICBT cannot adequately cover the entire tumor. The mean lymph node boost irradiation dose was significantly higher in the ICBT group. Cisplatin was the most commonly used systemic chemotherapy agent in both groups. HR-CTV D_90,_ D_95_ and rectum D_2cc_ in the HBT group received significantly higher doses than in the ICBT group, while bladder D_2cc_ showed no statistical difference.

**Table 2:** Treatment details

		ICBT (n = 280)	HBT (n = 189)	*P-*value
EBRT strategy				
	3D-CRT, WP + CS	263 (93.9%)	173 (91.5%)	0.003^*^
	3D-CRT, WP alone	17 (6.1%)	9 (4.8%)	
	IMRT	0 (0%)	7 (3.7%)	
Central Pelvic EBRT dose, (median, Gy)		30.6 (20.0-54.0)	30.0 (26.0-54.0)	0.602
LN boost (median, Gy)		0 (0-10)	6 (0-22.8)	0.001^*^
Systemic chemotherapy agents				
	CDDP	227 (81%)	152 (80.5%)	<0.001^*^
	CDDP +5-FU	0 (0%)	1 (0.5%)	
	CDDP + S-1	1 (0.4%)	0 (0%)	
	NDP	4 (1.4%)	7 (3.7%)	
	CBDCA	0 (0%)	1 (0.5%)	
	TP	29 (10.4%)	3 (1.6%)	
	Unknown	19 (6.8%)	25 (13.2%)	
No. of BT fractions				
	2 fractions	12 (4.3%)	1 (0.5%)	<0.001^*^
	3 fractions	52 (18.6%)	18 (9.5%)	
	4 fractions	211 (75.3%)	152 (80.5%)	
	5 fractions	5 (1.8%)	14 (7.4%)	
	6 fractions	0 (0%)	4 (2.1%)	
BT, EQD_2_ (a/b = 10, Gy)				
	HR-CTV D_90_ (median)	65.6 (41.3-102.0)	68.8 (49.4-97.3)	0.001^*^
	HR-CTV D_95_ (median)	60.6 (37.7-95.8)	65.0 (41.6-91.9)	<0.001^*^
BT, EQD_2_ (a/b = 3, Gy)				
	Rectum D_2cc_ (median)	51.4 (33.6-87.2)	58.4 (35.1-91.5)	<0.001^*^
	Bladder D_2cc_ (median)	65.7 (36.5-113.4)	68.4 (40.7-108.8)	0.178

EBRT: external beam radiation therapy

3D-CRT: 3-dimensional conformal radiation therapy

WP: whole pelvis

CS: central shield

IMRT: intensity modulated radiation therapy

LN: lymph node

CDDP: cisplatin

5-FU: 5-fluorouracil

S-1: an oral fluoropyrimidine

NDP: nedaplatin

CBDCA: carboplatin

TP: paclitaxel and carboplatin

BT: brachytherapy

EQD_2_: equivalent doses delivered in 2 Gy fractions

HR-CTV: high-risk clinical target volume

^*^Statistical significance was defined as a *P*-value of <0.05.

**Fig. 3.**
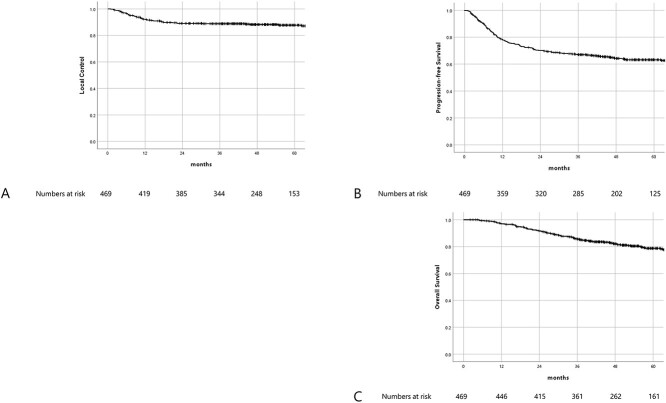
Shows Kaplan–Meier survival curves. Fig. 3A–C shows LC, PFS and OS, respectively.

### Efficacy

With a median follow-up of 51.3 months (2.1–139.9 months), 4-y LC, PFS and OS for the entire patient population were 88.2%, 64.2% and 83%, respectively (Fig. 3). Clinicopathological factors that influenced LC, PFS or OS are summarized in Table 3. In univariate analysis, histological subtype, uterine body invasion, tumor size before brachytherapy ≥4 cm, HR-CTV D_95_ ≤ 60 Gy, reduction ratio ≤ 29% and TTT ≥ 9 weeks were found to be negatively associated with LC. In multivariate analysis, non-Scc histological subtype, HR-CTV D_95_ ≤ 60 Gy, reduction ratio ≤ 29% and TTT ≥ 9 weeks were identified as the independent negative prognostic factors for LC. In univariate analysis, uterine body invasion, tumor size at diagnosis ≥7 cm, reduction ratio ≤ 29% and TTT ≥ 9 weeks were found to be negatively associated with PFS. In multivariate analysis, uterine body invasion, reduction ratio ≤ 29% and TTT ≥ 9 weeks were identified as the independent negative prognostic factors for PFS. In univariate analysis, uterine body invasion, tumor size before brachytherapy ≥4 cm, reduction ratio ≤ 29% and HBT were found to be negatively associated with OS. In multivariate analysis, uterine body invasion, reduction ratio ≤ 29% and HBT were identified as the independent negative prognostic factors for OS.

**Fig. 4.**
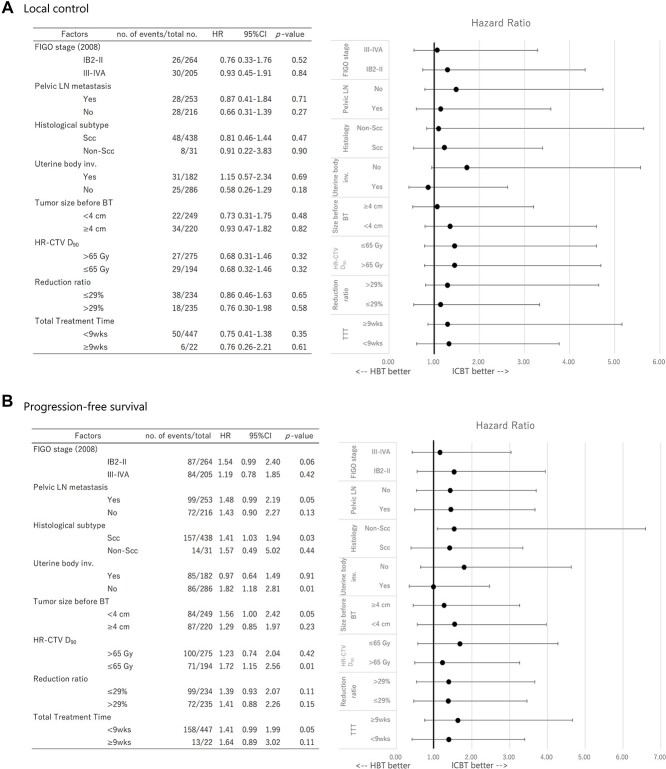

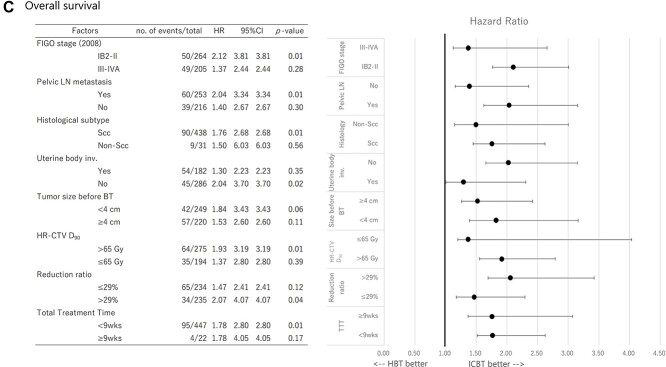
The forest plots show the results of Cox regression analysis regarding LC (4A), PFS (4B) and OS (4C) for ICBT and HBT.

**Fig. 5.**
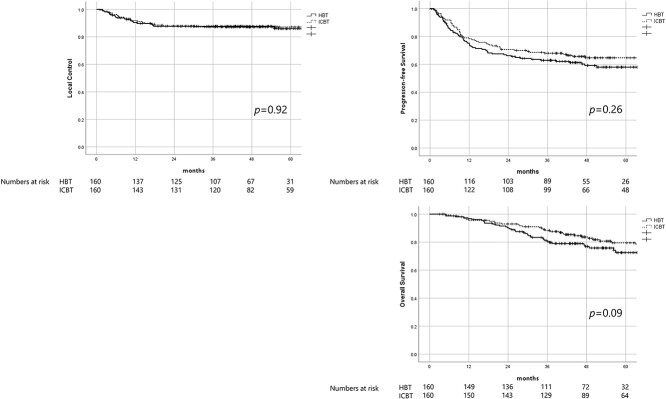
Shows the Kaplan–Meier LC, PFS and OS curves stratified by ICBT and HBT after adjustment of potential confounding factors between the two groups with propensity score matching.

The forest plots were drawn based on the results of Cox regression analysis regarding LC, PFS and OS between ICBT and HBT (Fig. 4). Results derived from the analysis showed no subgroup of patients benefited from HBT regarding LC, PFS and OS. To perform a statistical adjustment for pre-existing imbalance of baseline characteristics between the two groups, 1:1 propensity score matching was performed based on (confounding factors included were: stage, histology, uterine body invasion, tumor size before brachytherapy and reduction ratio). As a result, 320 patients (ICBT group: 160 patients, HBT group: 160 patients) were extracted. The matched-pair patients’ characteristics are summarized in Table 4. As shown in Table 4, no statistical difference was found between the two groups except the follow-up period. After adjustment of potential confounding factors between the two groups with propensity score matching, survival curves comparison was performed with the log-rank test regarding LC, PFS and OS (Fig. 5), however, no statistically significant difference was found between the two groups.

### Toxicities

A total of 29 patients (6.2%) experienced ≥ G3 late radiation-related toxicities. Table 5 summarizes the relationship between rectum or bladder D_2cc_ and late gastrointestinal (GI), genitourinary (GU) and vaginal toxicities. Unfortunately, the vaginal dose was not collected in this study. Instead, rectal dose was used as a surrogate marker for vaginal toxicities since it was thought that rectal dose may surrogate the vaginal dose because the ICRU recto-vaginal reference point is located on the anterior wall of the rectum [19]. While rectum D_2cc_ was associated with late GI and vaginal toxicities, bladder D_2cc_ was associated with late GU toxicities. Table 6 summarizes the relationship between the type of brachytherapy and late GI, GU and vaginal toxicities. While HBT was associated with increased late vaginal and ≥ G1 late GI toxicities, there was no difference in late ≥ G2 or ≥ G3, GI and GU toxicities.

## DISCUSSION

In this multi-national multi-institutional retrospective study involving Asian countries comparing the clinical outcomes of ICBT and HBT for locally advanced uterine cervical cancer, equivalent LC was observed in ICBT and HBT, despite patients treated with HBT having several adverse prognostic factors such as advanced stage, non-Scc, larger tumor size at diagnosis/before brachytherapy and a worse reduction ratio with statistical significance. To the best of our knowledge, this is the largest retrospective study comparing clinical outcomes between ICBT and HBT for Asian uterine cervical cancer patients.

In terms of LC, HBT could not demonstrate superiority to ICBT. Moreover, as shown in Fig. 4, the forest plots showed no subgroup of patients benefited from HBT regarding either LC, PFS, or OS. On the other hand, HBT was associated with a lower OS in this study, despite the fact that the mean lymph node boost dose in the HBT group was significantly higher (Table 1). It was presumably due to negative prognostic factors such as larger tumor size, advanced stage, poor reduction and uterine body invasion in patients included in the HBT group. To adjust such base-line imbalances between the two groups, propensity score matching analysis was performed using these prognostic factors; however, there was no statistical difference between HBT and ICBT in terms of LC, PFS and OS (Fig. 5). One possible explanation for this result is that the criteria for adapting HBT differed from institution to institution, and the indication of HBT could also have been changed during long the study period between 2000 and 2016 in the same institution, so it is possible that some patients who could have been cured even with ICBT were actually treated with HBT. Another possible reason why the advantage of HBT could not be clearly shown in this study was that the quality of HBT was not assessed and if any additional interstitial needles were used, the patient was categorized in the HBT group. As a result, it could be that patients treated by HBT may not always have been treated by brachytherapy with desirable dose distribution. Another possible reason could be that MRI, which is a gold standard image-guide modality of 3D-IGABT with superior tissue resolution to CT, was not used in most institutions included in this study and resulted in inadequate dose distribution, even though favorable clinical outcomes using CT-based 3D-IGABT with careful contouring, paying attention to the weakness of CT findings and taking account of recent MRI findings, have been reported. Although it is unclear which types of patients require HBT, some researchers attempted to answer this clinical question: Yoshida *et al.* [15] showed that HR-CTV size between 4 × 3 × 3 cm and 5 × 4 × 4 cm could be better treated by HBT, and Gonzalez *et al.* [20] found that tumor volume ≥ 35 cc or significant tumor asymmetry would benefit more from HBT. Despite the fact that the HBT group had more patients with advanced stage, uterine body invasion and parametrium invasion (Table 1) and rectum D_2cc_ was higher in HBT patients, bladder D_2cc_ was comparable between the two groups, suggesting that HBT is better than ICBT at delivering a higher dose to the target volume while sparing organs at risk (OAR). Taken together, despite the fact that HBT patients had more adverse clinical factors, HBT delivered a higher dose to HR-CTV and resulted in comparable LC. Thus, although the benefit of HBT was not clearly demonstrated after adjustment of imbalanced backgrounds with the propensity score matching analysis in this study, possibly because of uncollected imbalanced confounding factors that would influence clinical outcomes due to the nature of the retrospective study, actually HBT yielded comparable LC to ICBT despite the fact that the HBT group had more advanced clinical features than the ICBT group, additionally, other reports also have also shown favorable clinical results with HBT [12–14], HBT may be a promising and effective treatment method for locally advanced uterine cervical cancer.

In this study, the non-Scc histological subtype was associated with worse LC, which was consistent with previous reports [21, 22]. Furthermore, a tumor reduction ratio ≤ 29% was found to be a strong independent prognostic factor for LC, PFS and OS. Based on data from the international study on MRI-guided brachytherapy in locally advanced cervical cancer (EMBRACE) study, Jastaniyah *et al.* [23] clearly classified tumor response into six groups, and Mayr *et al.* [24] demonstrated in a retrospective analysis involving 115 patients that residual volumes at 40-50 Gy assessed by MRI were an independent prognostic factor for LC and OS. Minkoff *et al.* [22] also found that GTV at first brachytherapy >7.5 cc was associated with poorer 2-y LC, PFS and OS. These findings are consistent with our finding that tumor reduction ratio is an independent prognostic factor for uterine cervical cancer definitive radiotherapy.

**Table 3:** Hazard ratios for OS, LC and PFS in cervical cancer

Factors		Pts	OS		multivariate analysis		LC		multivariate analysis		PFS		multivariate analysis	
			4-y OS (%)	*P-*value	HR (95%CI)	*P-*value	4-y LC (%)	*P-*value	HR (95%CI)	*P-*value	4-y PFS (%)	*P-*value	HR (95%CI)	*P-*value
FIGO stage (2008)														
	IB2-II	264 (56.3%)	84.8	0.085			90.4	0.094			67.3	0.058		
	III-IVA	205 (43.7%)	78.3				85.3				60.0			
Pelvic LN metastasis														
	Yes	253 (53.9%)	80.6	0.125			88.9	0.571			62.4	0.111		
	No	216 (46.1%)	83.8				87.4				66.3			
Histological subtype														
	Scc	438 (93.4%)	82.8	0.056			89.2	0.007^*^	3.55 (1.66-7.60)	0.001^*^	65.1	0.119		
	Non-Scc	31 (6.6%)	72.1				73.6				50.9			
Uterine body inv.														
	Yes	182 (38.8%)	75.2	0.002^*^	0.53 (0.35-0.79)	0.002^*^	82.5	0.024^*^			52.7	0.001^*^	0.57 (0.42-0.76)	<0.001^*^
	No	286 (61%)	86.4				91.8				71.4			
	N/A	1 (0.2%)												
Pyometra														
	Yes	118 (25.2%)	80.9	0.719			69.7	0.566			57.4	0.309		
	No	351 (74.8%)	82.4				74.1				66.4			
Parametrium inv.														
	Yes	66 (14.1%)	82.2	0.070			71.5	0.209			63.1	0.252		
	No	403 (85.9%)	87.4				81.2				70.7			
Hydronephrosis														
	Yes	96 (20.5%)	84.3	0.654			74.4	0.663			61.2	0.554		
	No	373 (79.5%)	81.5				72.6				64.9			
Tumor size at diagnosis														
	<7 cm	386 (82.3%)	83.5	0.095			89.5	0.096			66.3	0.029^*^		
	≥7 cm	83 (17.7%)	75.5				82.7				54.5			
Tumor size before BT														
	<4 cm	249 (53.1%)	86.8	0.014^*^			91.3	0.021^*^			67.0	0.129		
	≥4 cm	220 (46.9%)	76.7				84.7				61.0			
HR-CTV D_90_														
	HR-CTV D_90_ > 65Gy	275 (58.6%)	81.4	0.229			90.5	0.080			64.5	0.707		
	HR-CTV D_90_ ≤ 65Gy	194 (41.4%)	83.1				85.0				63.8			
HR-CTV D_95_														
	HR-CTV D_95_ > 60Gy	269 (57.4%)	80.9	0.099			90.3	0.020^*^	2.14 (1.25-3.64)	0.005^*^	63.9	0.076		
	HR-CTV D_95_ ≤ 60Gy	181 (38.6%)	82.1				83.7				62.0			
	Unknown	19 (4%)												
Reduction ratio														
	≤29%	234 (49.9%)	74.5	<0.001*	2.02 (1.33-3.07)	0.001^*^	84.0	0.004^*^	2.49 (1.41-4.38)	0.002^*^	58.2	0.010^*^	1.49 (1.1-2.0)	0.010^*^
	>29%	235 (50.1%)	89.6				92.4				70.2			
Total Treatment Time														
	<9wks	447 (95.3%)	82.1	0.943			88.9	0.021^*^	0.33 (0.17-0.93)	0.033^*^	65.2	0.021*	0.56 (0.32-0.98)	0.042^*^
	≥9wks	22 (4.7%)	81.0				73.5				43.8			
Type of BT														
	ICBT	280 (59.7%)	85.3	0.004^*^	1.51 (1.00-2.27)	0.049^*^	89.3	0.314			68.4	0.014^*^		
	HBT	189 (40.3%)	77.0				86.8				57.8			

FIGO: the International Federation of Gynecology and Obstetrics

LN: lymph node

BT: brachytherapy

HR-CTV: high-risk clinical target volume

ICBT: intracavitary brachytherapy

HBT: hybrid brachytherapy

OS: overall survival

LC: local control

PFS: progression-free survival

^*^Statistical significance was defined as a *P*-value of <0.05.

**Table 4:** Patient characteristics (patched-pair)

		ICBT (*n* = 160)	HBT (n = 160)	*P-*value
Age (median), years		57 (28-86)	58 (26-81)	0.942
Follow-up period (median), months		50.8 (5.2-138.4)	45.5 (3.0-105.3)	<0.001^*^
FIGO stage (2008)				
	IB2-II	73 (45.6%)	72 (45%)	1
	III-IVA	87 (54.4%)	88 (55%)	
Histological subtypes				
	Squamous cell carcinoma	149 (93.1%)	150 (93.8%)	0.226
	Adenocarcinoma	6 (3.8%)	9 (5.6%)	
	Adenosquamous carcinoma	5 (3.1%)	1 (0.6%)	
Uterine body invasion				
	Yes	59 (36.9%)	77 (48.1%)	0.054
	No	101 (63.1%)	83 (51.9%)	
Pyometra				
	Yes	45 (28.1%)	35 (21.9%)	0.198
	No	115 (71.9%)	125 (78.1%)	
Parametrium invasion				
	Yes	148 (92.5%)	143 (89.4%)	0.223
	No	12 (7.5%)	17 (10.6%)	
Hydronephrosis				
	Yes	33 (20.6%)	40 (25%)	0.221
	No	127 (79.4%)	120 (75%)	
Pelvic LN metastasis				
	Yes	89 (55.6%)	88 (55%)	1
	No	71 (44.4%)	72 (45%)	
Tumor size at diagnosis (median, cm)		5.9 (4.0-12)	5.7 (4.1-11)	0.835
Tumor size before brachytherapy (median, cm)		4.1 (1.6-6.6)	4.1 (2.0-9.0)	0.087
Reduction ratio (%)		29 (0-74)	28 (0-71)	0.084
Total treatment time (median, weeks)		7 (5-11)	7 (5-14)	0.914

FIGO: the International Federation of Gynecology and Obstetrics

LN: lymph node

ICBT: intracavitary brachytherapy

HBT: hybrid brachytherapy

^*^Statistical significance was defined as a *P*-value of <0.05.

While HR-CTV D_90_ is a well-recognized parameter that predicts LC [2, 5, 21, 23, 25], HR-CTV D_95_ was found to be a better predictor of LC in this study. D_90_ ignores 10% of the target volume, whereas D_95_ ignores only 5%, and this difference could be translated into a significant difference when applied to the large tumors. And the absolute volume of this ignored volume would be larger when the tumor is larger. Therefore, it could be said that when the ignored percentage is smaller, such influence will become smaller, especially for larger tumors. Okazaki *et al.* [26] reported that, in addition to HR-CTV D_90_, HR-CTV D_98_ was associated with tumor control. Therefore, HR-CTV D_X_ greater than D_90_ may thus be a better surrogate dose-volume parameter for LC. In this study, HR-CTV D_95_ > 60 Gy was found to be associated with better LC. Although 60 Gy is much lower than the doses written in the guidelines from the Gynaecological Groupe Europeen de Curietherapie-European Scoiety for Radiotherapy & Oncology (GEC-ESTRO) [1] or American Brachytherapy Society (ABS) [7], the dose contribution from CS was not considered in the calculation due to the complexity [17], and the dose contribution to HR-CTV D_90_ from CS with 4 cm width being 13–35% depending on the size of the tumor [27], so the actual dose delivered to the target would have been higher than 60 Gy. CS has the advantage of being able to easily reduce the rectal and bladder doses. However, this technique has a significant disadvantage in that the actual delivered dose may be difficult to assess unless complicated image registration is not performed. If our society continues to use CS, attempting to obtain the actual delivered dose should be assessed precisely in some practical and simple ways.

In this study, uterine body invasion was found to be an unfavorable prognostic factor, which was consistent with the previous report [14]. The current emphasis on incorporating interstitial needles is primarily on covering lateral tumor spread. However, when there is severe asymmetric uterine body invasion that cannot be adequately covered by tandem, it may be encouraged that interstitial needles be inserted to supplement the uterine body tumor asymmetry. When LC was compared between ICBT and HBT for 182 patients only with uterine body invasion, no statistically significant difference was found (4-y LC 81% vs 84.3%, *P* = 0.691), indicating that the current cohort of patients’ intent for using HBT was not primarily to cover the uterine body invasion. Interstitial needle insertion guided by a combination of transrectal and transabdominal ultrasonography would be an appropriate technique to navigate needle position for deeply situated tumors such as uterine body invasion in real-time fashion [28].

TTT is an established prognostic factor for uterine cervical cancer [29, 30]. In addition to these previous reports, TTT ≥ 9 weeks was found to be an independent negative prognostic factor in this study. However, because HBT requires more expertise and labor than conventional ICBT, it is conceivable that longer TTT is required when referring patients to a distant institution capable of HBT. As a result, such a logistical issue should be addressed, with at least one institution in a large medical area offering HBT.

Although mean rectum D_2cc_ was higher in HBT than ICBT (Table 2), no statistical difference was found concerning late ≥ G2 toxicities between groups (Table 6). If higher HR-CTV doses were due to simply allowing a higher dose, both the rectal and bladder dose should have been increased. In reality, this was not the case (Table 2). Thus, with HBT, higher HR-CTV D_90_ and D_95_ doses (Table 2) were delivered with equivalent late GI toxicities, which is a significant advantage for using HBT for locally advanced cervical cancer. Nonetheless, as previously reported [31, 32], rectum D_2cc_ and bladder D_2cc_ were associated with late rectal and urogenital toxicities (Table 5). According to the findings of this study, non-Scc histology or a reduction rate ≤ 29% were associated with worse LC. For such unfavorable tumors, dose escalation is required to improve clinical outcomes. Therefore, when escalating target doses to ensure LC, doses to the surrounding normal OARs should be kept as low as possible. As shown in the treatment of prostate cancer [33], gel spacers may be useful in lowering OAR doses in the management of gynecologic malignancies [32, 34–37]. Rectum D_2cc_ was also associated with late vaginal toxicities, lending credence to the ICRU rectovaginal reference point, which is located at the intersection of the tandem and vaginal source positions and 5 mm dorsal of the posterior vaginal wall [19].

Although there was no statistical difference ≥ G3 vaginal toxicities between the two groups, the HBT group had more ≤ G2 vaginal toxicities (Table 6). Because no data on vaginal doses or interstitial needle pathways were collected in the current study, no confirmatory description can be made. However, higher vaginal doses or needle insertion through the vaginal wall could have contributed to the higher incidence of G2 vaginal toxicities in the HBT group.

The majority of the patients in this study were treated with CS to protect OARs such as the rectum and the bladder, which is obviously not a standard treatment strategy in the United States or most European countries [1, 6]. Therefore, even if 13–35% of a CS dose was actually delivered to the target volume as mentioned before, the median HR-CTV D_90_ in this study was lower than the recommended HR-CTV D_90_ of 85 Gy EQD_2_ by the GEC-ESTRO. Recently, results of EMBRACE-I, a large multi-institutional prospective observational study involving ≥1300 patients, have been reported [38], in which as much as ≥90% of 5-year LC was consistently achieved regardless of T stage. On the other hand, up to 14.6% of patients experienced grade 3–5 late toxicities, rising to 18.4% when focusing on Stage III-IVA, which is unacceptable to our society, and this high late toxicity rate is the reason why our society’s guideline recommendation still does not adopt 85 Gy EQD_2_ as a prescription goal. Because the majority of uterine cervical cancer patients are related to the human papillomavirus (HPV) and similar to the HPV-related oropharyngeal cancer [39, 40], de-intensification for uterine cervical cancer could be considered as demonstrated by a subgroup analysis of the current patient cohort [41]. Even if IMRT is used for EBRT, a 1–2 cm margin is required to compensate for internal uterine motion, resulting in exposure to the surrounding bowel. Although it is understandable that some patients with radio-resistance require a higher dose of >85 Gy EQD_2_, it is unsafe to administer a high dose to all patients regardless of tumor response due to the high rate of late radiation-related toxicities. In this context, CS may be a viable option for effectively and easily reducing OAR doses, resulting in a lower rate of late severe toxicities, as shown in this retrospective study, while maintaining reasonable LC.

This study has a number of limitations. Because this was a retrospective study, the treatment protocols across the participating institutions differed, including EBRT dose, technique, contouring guidelines, imaging protocol, number of brachytherapy fractions, usage of inhomogeneity correction in brachytherapy dose calculation, or systemic chemotherapy. Furthermore, the definition of an indication for applying HBT varied between institutions; therefore, tumors that could also be treated with ICBT were potentially treated with HBT. There was selection bias in deciding whether to use ICBT or HBT, and as shown in Table 1, more advanced patients were more likely to be treated with HBT. Contouring uncertainty in IGABT, which potentially plays a significant role in dose-volume histogram parameters such as D_90_ and D_95_, was not addressed in the study. Important information such as HR-CTV volume prior to treatment and at the time of brachytherapy was missing. Despite these limitations listed above, along with recent studies supporting the efficacy of HBT [12–14], the current study, with an adequate number of patients and a long follow-up period, supports the efficacy of HBT in treating locally advanced uterine cervical cancer. However, since the follow-up period in the HBT group was shorter than that of the ICBT group (Table 1), it has to be said that the results derived from this study are inconclusive.

**Table 5:** Relationship between rectal and bladder dose and late GI, vaginal and GU toxicity

	Late GI toxicity ≥ G1			Late GI toxicity ≥ G2			Late GI toxicity ≥ G3		
	Yes (*n* = 130)	No (*n* = 339)	*P*-value	Yes (*n* = 48)	No (*n* = 421)	*P*-value	Yes (*n* = 17)	No (*n* = 452)	*P*-value
Mean total rectum D_2cc_ (EQD_2_, α/β = 3, Gy)	58.8	53.2	<0.001^*^	59.6	54.2	0.001^*^	61.9	54.5	0.007^*^
	Late Vaginal toxicity ≥ G1			Late Vaginal toxicity ≥ G2			Late Vaginal toxicity ≥ G3		
Mean total rectum D_2cc_ (EQD_2_, α/β = 3, Gy)	Yes (*n* = 53)	No (*n* = 416)	*P*-value	Yes (*n* = 15)	No (*n* = 454)	*P*-value	Yes (*n* = 8)	No (*n* = 461)	*P*-value
	59.6	54.2	<0.001^*^	59.3	54.7	0.111	59.1	54.7	0.266
	Late GU toxicity ≥ G1			Late GU toxicity ≥ G2			Late GU toxicity ≥ G3		
Mean total bladder D_2cc_ (EQD_2_, α/β = 3, Gy)	Yes (*n* = 77)	No (*n* = 392)	*P*-value	Yes (*n* = 36)	No (*n* = 433)	*P*-value	Yes (*n* = 9)	No (*n* = 460)	*P*-value
	71.2	64.1	<0.001^*^	73.4	64.6	0.001^*^	72.3	65.1	0.155

EQD_2_: equivalent doses delivered in 2 Gy fractions

GI toxicity: gastrointestinal toxicity

GU toxicity: genitourinary toxicity

^*^Statistical significance was defined as a *P*-value of <0.05.

**Table 6:** **.** Relationship between type of brachytherapy and late GI, vaginal and GU toxicity

Type of Brachytherapy	Late GI toxicity ≥ G1	*P*-value	Late GI toxicity ≥ G2	*P*-value	Late GI toxicity ≥ G3	*P*-value
ICBT (280)	67 (23.9%)	0.026^*^	28 (10%)	0.838	10 (3.6%)	0.940
HBT (189)	63 (33.3%)		20 (10.6%)		7 (3.7%)	
	Late Vaginal toxicity ≥ G1		Late Vaginal toxicity ≥ G2		Late Vaginal toxicity ≥ G3	
ICBT (280)	18 (6.4%)	<0.001^*^	5 (1.8%)	0.034^*^	4 (1.4%)	0.412
HBT (189)	35 (18.5%)		10 (5.3%)		4 (2.1%)	
	Late GU toxicity ≥ G1		Late GU toxicity ≥ G2		Late GU toxicity ≥ G3	
ICBT (280)	45 (16.1%)	0.805	24 (8.6%)	0.375	4 (1.4%)	0.271
HBT (189)	32 (16.9%)		12 (6.3%)		5 (2.6%)	

ICBT: intracavitary brachytherapy

HBT: hybrid brachytherapy

GI toxicity: gastrointestinal toxicity

GU toxicity: genitourinary toxicity

^*^Statistical significance was defined as a *P*-value of <0.05.

## CONCLUSION

Despite the fact that HBT patients had more adverse clinical factors than ICBT patients, HBT delivered a higher dose to HR-CTV and resulted in comparable LC, even though clear superiority of HBT over ICBT could not be shown after adjusting for potential confounding clinical factors with propensity score matching. HBT may be a promising treatment method for patients with locally advanced uterine cervical cancer.

### ACKNOWLEDEGMENT

The authors are grateful for all doctors who were involved in data collection for this retrospective study. The authors would also like to express their heartful gratitude to Ryunosuke Machida, a biostatistician, for providing us with valuable and insightful advice on the statistical analyses of this article. Part of the patients’ data was provided from institutions involved in the Working Group of the Gynecological Tumor Committee of the Japanese Radiation Oncology Study Group (JROSG).

## CONFLICT OF INTEREST

Dr. Itami reports personal fees from HekaBio, grants and personal fees from Itochu, grants from Elekta, personal fees from AlphaTAU, personal fees from ViewRay, personal fees from Palette Science, outside the submitted work.

Dr. Igaki reports personal fees from HekaBio, personal fees from AstraZeneca, personal fees from Itochu, outside the submitted work.

This study receives no financial support from any company, so there are no conflicts of interests to declare.

## FUNDING

This study was partially supported by The Japan Agency for Medical Research and Development (AMED, 19ck0106305h0003) and the National Cancer Center Research and Development Fund (26-A-18 and 26-A-28).

## ETHICAL STATEMENT

All researchers involved in this study acted and performed the research according to the Declaration of Helsinki and Ethical guidelines for medical and health research involving human subjects. The research started after receiving approval from the local institutional ethical review board (the approval number was 2018-245). Because of the retrospective and observational nature of this study, the requirement for written informed consent was waived on the condition that a document declaring an opt-out policy by which any potential patient and/or relatives could refuse to be included in this study was uploaded to the National Cancer Center Hospital Web page. Under the philosophy of individual respect for personality, information that can identify a person was anonymized and the collected data was securely stored and handled in accordance with the regulations.
